# Designed Omnidirectional Antenna of Quarter-Mode Substrate-Integrated Waveguide Element with Characteristic Mode Analysis

**DOI:** 10.3390/mi16060717

**Published:** 2025-06-17

**Authors:** Wei Hu, Liangfu Peng, Tao Tang, Maged A. Aldhaeebi, Thamer S. Almoneef, Jaouhar Mouine

**Affiliations:** 1 College of Electronic and Information, Southwest Minzu University, Chengdu 610225, China; huwei@swun.edu.cn (W.H.); 21500072@swun.edu.cn (L.P.); 2 Department of Electrical Engineering, Prince Sattam Bin Abdulaziz University, Al-Kharj 11942, Saudi Arabia; m.aldhaeebi@psau.edu.sa (M.A.A.); t.almoneef@psau.edu.sa (T.S.A.); j.mouine@psau.edu.sa (J.M.)

**Keywords:** omnidirectional antenna, low-profile, characteristic mode analysis, substrate-integrated waveguide

## Abstract

This study investigates the design of omnidirectional antennas, using a characteristic mode analysis (CMA), and explores two distinct feeding methods. The first method employs equal-amplitude and in-phase excitation across all ports, whereas the second method utilizes equal-amplitude excitation with a 180° phase difference between adjacent ports. Both designs achieve operating bandwidths of 2.45–2.58 GHz and 2.42–2.45 GHz, respectively, with peak gains of 4.1 dBi and 4.4 dBi at 2.45 GHz. The proposed antennas exhibited high gain and low-profile characteristics, making them well-suited for applications in wireless energy harvesting.

## 1. Introduction

Omnidirectional antennas are essential for transmitting and receiving electromagnetic wave signals in all directions, with widespread applications in wireless local area networks (WLAN), Bluetooth, and other communication systems [1,2]. However, conventional omnidirectional antenna designs, which are primarily based on monopole antennas, often exhibit high-profile characteristics that limit their integration into compact low-profile systems. For instance, traditional monopole antennas typically have a height of λ/4, which restricts their practical applications with their high profile [3]. This limitation has led to an increasing demand for low-profile, small, and compact omnidirectional antennas that can meet modern integration requirements [4].

The implementation of omnidirectional antennas can be broadly categorized into two approaches: single antennas [5,6,7,8,9] and array antenna structures [10,11,12,13,14,15,16]. Modified patch antenna structures such as slot structures and periodic metal probes enable operation in zero- or high-order modes, achieving quasi-omnidirectional patterns similar to those of monopole antennas. For example, Liu et al. demonstrated a circular patch antenna loaded with a metal probe, operating in TM01/TM02 modes, with a diameter of 180 mm (1.44 λ_0_) and thickness of 0.024 λ_0_, and achieving a peak gain of 6 dBi at 2.45 GHz [5]. Further enhancements involved etching the ring structure in the circular radiation layer and adding a metal probe, allowing operation in the TM01 and TM02 modes with broadband omnidirectional characteristics. The enhanced antenna measured 120 mm (2.4 λ_0_) in diameter and 0.029 λ_0_ in thickness, while maintaining a peak gain of 6.0 dBi [6].

Al-Zoubi etched the ring structure in a circular patch and obtained omnidirectional characteristics when the antenna operated in the TM01 mode [7]. The antenna had a diameter of 150 mm (3 λ_0_), thickness of 1.5 mm (0.03 λ_0_), and peak gain of 5.7 dBi at a working frequency of 5.8 GHz. Park proposed the use of a negative dielectric constant transmission line to achieve circular polarization and omnidirectional patterns in zero- and first-order modes [8]. Their design reported a gain of −0.24 dBic for the zero-order mode and −0.51 dBic for the first-order mode of right-hand circular polarization.

These approaches highlight the potential of higher-order modes such as TM22 for achieving omnidirectional characteristics in directional patterns [9]. Although these designs offer lower profile heights and simplify antenna processing and assembly, they result in larger structures and radiation layers. Additionally, this approach typically exhibits a lower gain, requiring larger dielectric or air layers to achieve a higher gain [10].

Array-based omnidirectional antennas, on the other hand, utilize element configurations such as 2 × 2, 4 × 4, etc., with each element excited by equal-amplitude and in-phase signals to achieve symmetrical electric field distributions and omnidirectional patterns [11,12,13,14,15,16]. For example, Yu et al. employed an arc-shaped printed dipole antenna with a 1:4 power division network, achieving a gain of 4.0 dBi at 2.45 GHz [11]. Similarly, Gao et al. utilized a Vivaldi unit structure with strong mutual coupling effects between 16 antenna units, resulting in a high gain and broad bandwidth. Sun et al. designed a 2 × 2 array using bent dipole elements to achieve omnidirectional patterns through equal amplitude and phase excitations [12].

The characteristic mode theory (CMT) offers a novel approach for analyzing antenna working modes [17,18,19]. Gong et al. designed a loading antenna based on a metasurface structure, using the eigenmode theory to analyze various working modes and radiation patterns, which enabled the achievement of omnidirectional radiation patterns using a slot-coupled method [18]. Further applications of CMT of metasurface antennas demonstrated that different directional patterns could be achieved by adjusting excitation signals, with 90° phase differential producing circularly polarized patterns [19].

A comparative analysis of these designs revealed that an omnidirectional pattern with high gain was achieved by exciting each antenna unit, using signals of equal amplitude and phase. However, this approach operates at a fundamental mode. In the design analysis and process, the mutual coupling effects between adjacent antenna units must be reduced to ensure optimal performance. While increasing the element spacing reduces the coupling, it also leads to a larger overall size, which hinders compact integration.

To address these limitations, this study uses the characteristic mode theory to investigate the working principle of a quarter-mode substrate-integrated waveguide (SIW) structure as a unit antenna used to achieve omnidirectional radiation patterns. Two implementation methods are proposed: (1) exciting each antenna unit with signals of equal amplitude and phase, and (2) exciting adjacent antenna units with signals of equal amplitude but a 180° phase difference, following the sequence (0°, 180°, 0°, 180°).

The key innovation of this study is the design of an omnidirectional antenna based on a quarter-mode unit structure, leveraging the characteristic-mode theory for performance analysis. Through surface current analysis, a feed power subnetwork with a (0°, 180°, 0°, 180°) configuration was designed to enhance omnidirectional characteristics. This method is a novel implementation strategy.

## 2. Design Process of Antenna Unit Structure

### 2.1. Antenna Design Process

**Figure 1:**
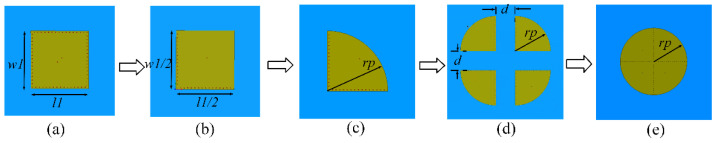
Design process for the omnidirectional antenna: (**a**) SIW; (**b**) quarter-mode SIW; (**c**) circular quarter-mode SIW; (**d**) 2 × 2 array circular quarter-mode SIW; and (**e**) the antenna design.

The design process of the proposed omnidirectional antenna is illustrated in Figure 1. The antenna unit was based on a quarter-mode SIW structure [20,21,22], as shown in Figure 1a–c. This unit forms a 2 × 2 array configuration, as depicted in Figure 1d, with an inter-element spacing of d (d < 0.5 λ_0_). A metal, cylinder-shaped structure was introduced between adjacent antenna units to minimize mutual coupling. When the spacing between the antenna units is reduced to zero, the four units are integrated into a single structure, as shown in Figure 1e. The dimensions of SIW with l1 = w1 = 82 mm, the radius of the antenna radiation structure is rp = 41 mm, the thickness of dielectric substrate is 3.0 mm, and the size of the antenna is 82 mm × 82 mm.

According to the theory of quarter-mode SIW (QHSIW) structure, the initial parameters of the antenna structure can be obtained. The resonance frequency of the SIW resonator can be calculated according to the following, Formula (1):

(1) $$\begin{array}{l} f_{mn0}=\frac{c}{2\sqrt{\varepsilon_{r}}}\sqrt{{\left(\frac{m}{L_{eff}}\right)}^{2}+{\left(\frac{n}{W_{eff}}\right)}^{2}}\\ L_{eff}=L_{p}-\frac{d^{2}}{0.95s}\\ W_{eff}=W_{p}-\frac{d^{2}}{0.95s} \end{array}$$

where *c* is the speed of light in free space; $\varepsilon_{r}$ is the relative dielectric constant of the medium; m, n are the mode numbers of SIW; $L_{eff}$ is the equivalent length; and $W_{eff}$ is the equivalent width of the SIW cavity structure.

With the requirements of the SIW design, the diameter of the via holes *d*, and the spacing between the via holes *s*, meet condition with Formula (2):

(2) $$\left\{\begin{array}{l} d\leq \frac{\lambda }{10}\\ s\leq 2d \end{array}\right.$$

**Figure 2:**
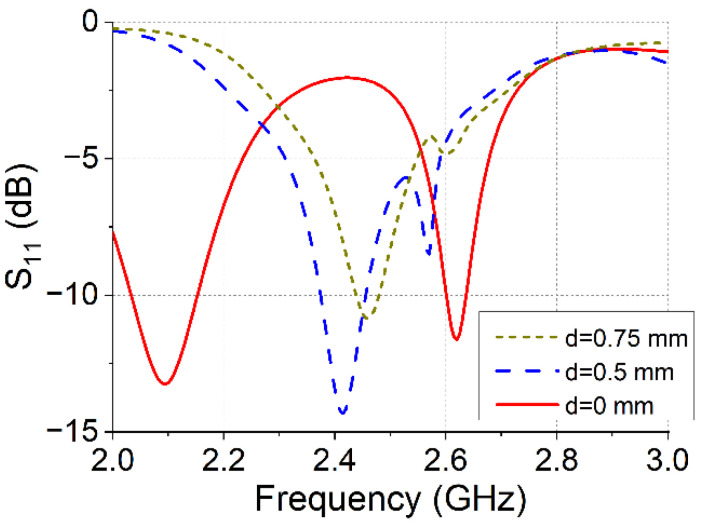
S_11_ for different antenna-spacing distances.

A 2 × 2 array configuration was examined to analyze the performance of the designed antenna structure. Figure 2 shows the simulated S_11_ results for different antenna-spacing distances.

As the spacing between the units increases, the mutual coupling weakens, resulting in a gradual increase in the coupling coefficient. However, the primary focus is on the shift in the resonance frequency between the ports as the distance changes. A comparative analysis revealed that integrating the four antenna units effectively increased the overall size of the antenna, leading to a lower resonant operating frequency. Under the same structural size conditions, the proposed antenna design achieved a more compact form with reduced dimensions and without antenna element spaces, offering significant advantages in terms of size and integration.

### 2.2. Antenna Mode Analysis Using Characteristic Mode Analysis (CMA)

The characteristic mode provides a new method for analyzing the distribution characteristics of the antenna mode, and modal significance is one of the more important parameters in the characteristic mode analysis method. The correspondence is shown in Formula (3).

(3) $$MS=\left.\left|\frac{1}{1+j\lambda_{n}}\right.\right|$$

$\lambda_{n}$ is eigenvalue; the value of MS ranges from 0 to 1; and when the value of the mode importance is 1, the corresponding eigenvalue is 0, which also indicates that the radiation structure is in a resonant state at the working frequency, and the corresponding frequency is the working frequency of the radiation structure.

The characteristic angle describes the phase difference between the surface current distribution in each mode and the surface electric field generated by the current distribution. The characteristic angle $\beta_{n}$ is shown in Formula (4).

(4) $$\beta_{n}=180^{{}^{\circ}}-\arctan\lambda_{n}$$

**Figure 3:**
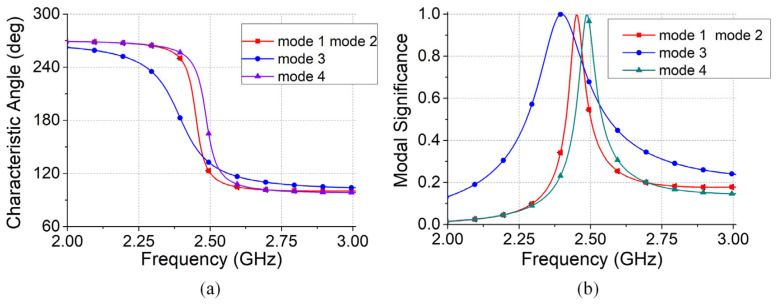
Antenna structure characteristic mode distribution: (**a**) characteristic angle; (**b**) characteristic mode.

The CMA module in the CST software 2023 was employed to facilitate analysis of the omnidirectional radiation pattern. Figure 3 illustrates the characteristic angle and mode distribution of the antenna. Within the operating frequency range, the antenna exhibited four distinct modes. Modes 1 and 2 coincide at the same operating frequency, whereas Mode 4 operates at around 2.45 GHz.

**Figure 4:**
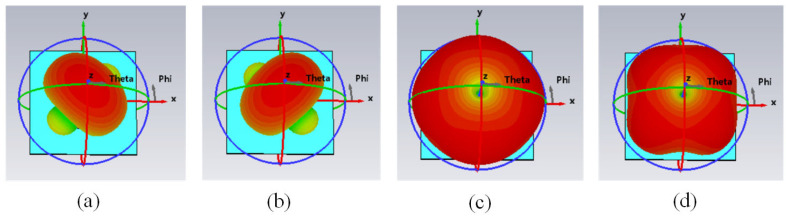
Antenna modes 1, 2, 3, and 4 in the far-field pattern: (**a**) Mode 1; (**b**) Mode 2; (**c**) Mode 3; and (**d**) Mode 4.

The far-field radiation patterns and surface current distributions corresponding to modes 1, 2, 3, and 4 are shown in Figure 4. The multiple-mode distribution of the proposed antenna was analyzed using the CMA. The distribution characteristics of the different modes confirmed that the antenna achieved an omnidirectional radiation pattern.

**Figure 5:**
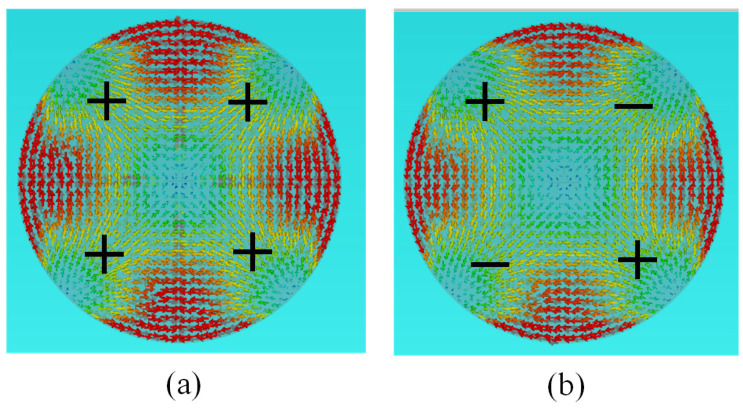
Surface current distribution of the antenna: (**a**) equal amplitude with in-phase; (**b**) equal amplitude with out-phase.

The surface current distribution diagram of the antenna (Figure 5) shows that when the excitation ports operate with equal amplitudes and a 180° phase difference between adjacent ports, the antenna exhibits central symmetry and achieves an omnidirectional pattern.

According to the distribution relationship of the antenna surface current, when the same amplitude and in-phase feed were adopted, the antenna exhibited omnidirectional characteristics. Similarly, when the phase of the excitation signal meets the (0°, 180°, 0°, 180°) excitation mode, the antenna can also realize an omnidirectional directional pattern. Characteristic mode theory is used to analyze the radiation-based characteristics of the antenna, and this provides a reference for subsequent antenna research and design.

The multiple-mode distribution of the proposed antenna was analyzed using the CMA method. The distribution characteristics of different modes confirmed that the antenna achieved an omnidirectional radiation pattern.

## 3. Design of Array Antenna I

**Figure 6:**
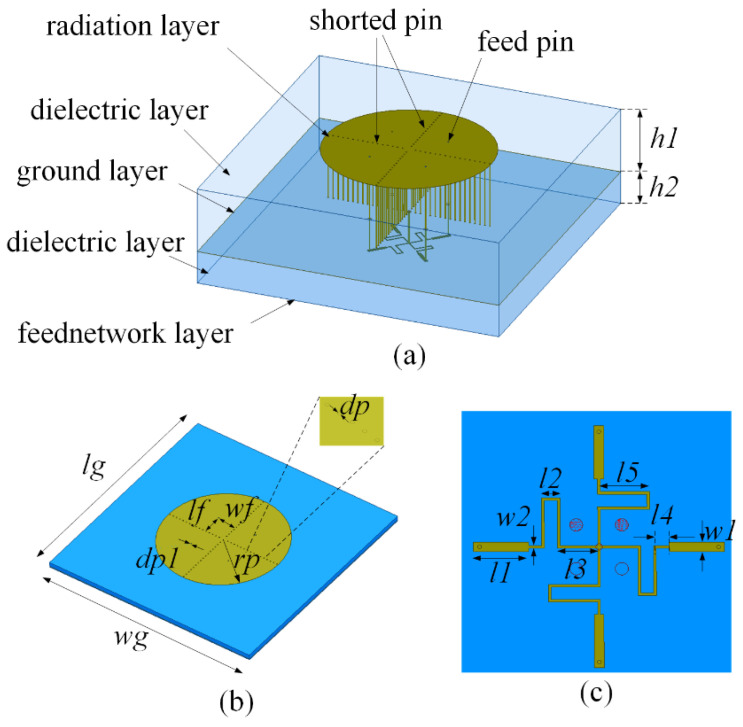
Configuration of the antenna structure: (**a**) 3D structure; (**b**) patch; and (**c**) feed network.

Based on the characteristic modes and far-field radiation patterns, a power distribution network was designed, with the aim of achieving an omnidirectional radiation pattern. The proposed antenna structure consists of multiple layers, including the upper radiation, intermediate dielectric, lower metal ground, feed network dielectric, and feed signal layers, as shown in Figure 6.

The upper dielectric layer used an F4BM220 material with a dielectric constant of 2.2, tangent loss of 0.001, and thickness of 3.0 mm. The specific dimensions of the antenna structure which is detailed in Figure 6 are as follows: lg = 140 mm, wg = 140 mm, rp = 38.3 mm, lf = 13 mm, wf = 13 mm, dp = 0.6 mm, dp1 = 0.6 mm, l1 = 8.5 mm, l2 = 2.82 mm, l3 = 6.2 mm, l4 = 2 mm, l5 = 7.59 mm, w1 = 1.5 mm, w2 = 0.41 mm, h1 =3 mm, and h2 = 0.508 mm.

**Figure 7:**
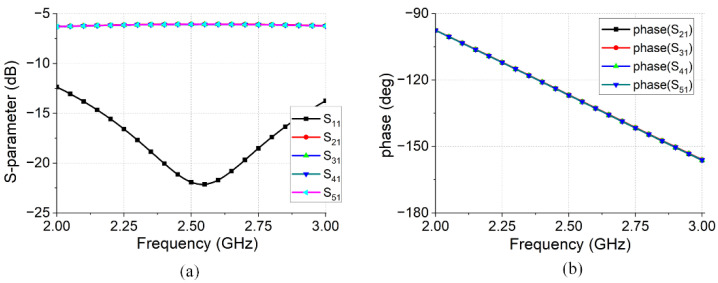
Simulation results for the feed network: (**a**) amplitude; (**b**) phase.

The feed network structure shown in Figure 6b features a central input port and four surrounding output ports (2–5). The simulation results for the feed network are presented in Figure 7. The simulation results showed equal amplitudes within the range of 2.0–3.0 GHz. At 2.45 GHz, the insertion loss is 0.07 dB, indicating a low insertion loss. In addition, the phases of each output port are uniform, satisfying the requirements for in-phase excitation.

**Figure 8:**
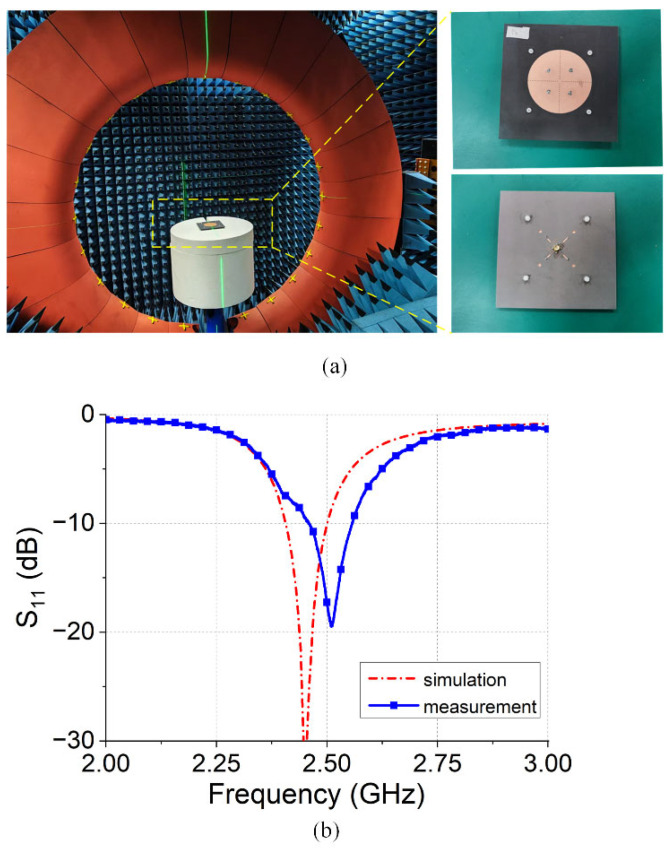
Antenna measurement: (**a**) measurement platform; (**b**) S_11_.

**Figure 9:**
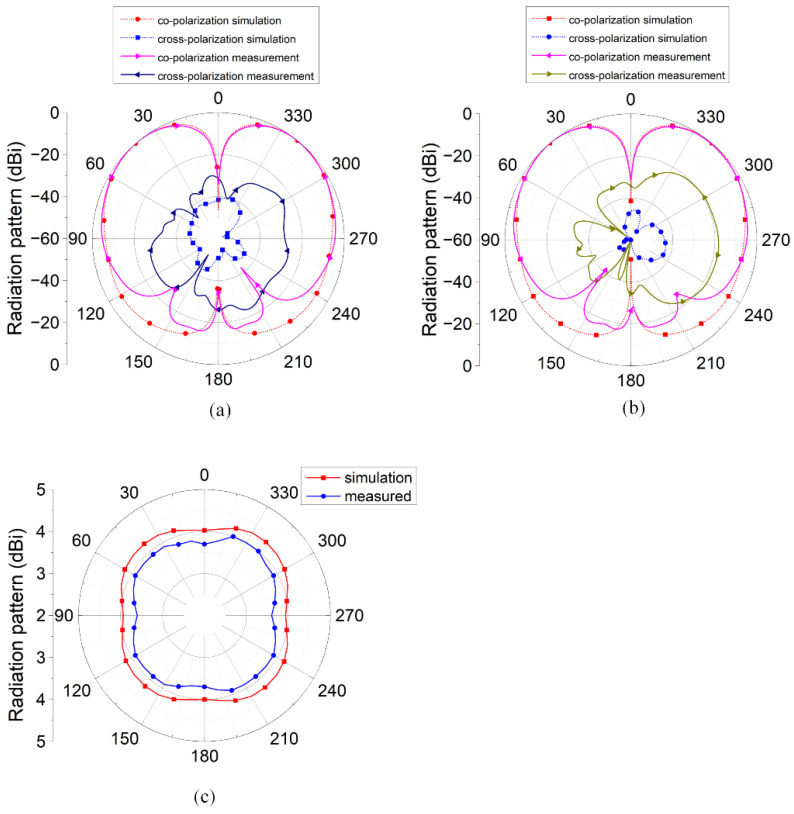
Radiation pattern of the antenna: (**a**) XOZ plane; (**b**) YOZ plane; and (**c**) peak gain at theta = 39°.

The antenna measurement results and the simulation results are shown in Figure 8 and Figure 9, respectively. The −10 dB bandwidth of the antenna is 2.45–2.58 GHz (130 MHz bandwidth), with an S11 value of −10.5 dB at 2.45 GHz. Figure 9 shows the radiation patterns of the antennas. At 2.45 GHz, the antenna exhibited omnidirectional radiation characteristics in both the XOZ and YOZ planes, achieving a peak gain of 4.1 dBi at theta = 39°.

## 4. Design of Array Antenna II

**Figure 10:**
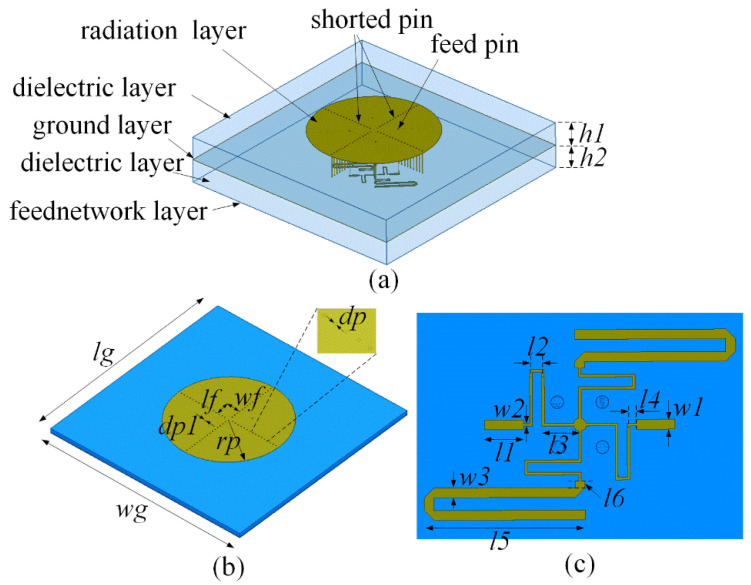
Antenna structure: (**a**) 3D structure; (**b**) patch; and (**c**) feed network.

The second proposed omnidirectional antenna design is based on a similar quarter-mode SIW structure, but with a different feeding strategy. The antenna structure is shown in Figure 10. The radiation layer used was F4BM220 with a thickness of 3.0 mm, and the feed network layer also employed F4BM220 with a thickness of 0.508 mm.

The specific dimensions of the antenna structure are detailed in Figure 10, as follows: lg = 150 mm, wg = 150 mm, rp = 39.65 mm, lf = 10 mm, wf = 10 mm, dp = 0.6 mm, dp1 = 0.6 mm, l1 = 6 mm, l2 = 1.89 mm, l3 = 5.5 mm, l4 = 1.18 mm, l5 = 25 mm, l6 = 1.0 mm, w1 = 1.5 mm, w2 = 0.39 mm, w3 = 1.5 mm, h1 = 3 mm, and h2 = 0.508 mm.

**Figure 11:**
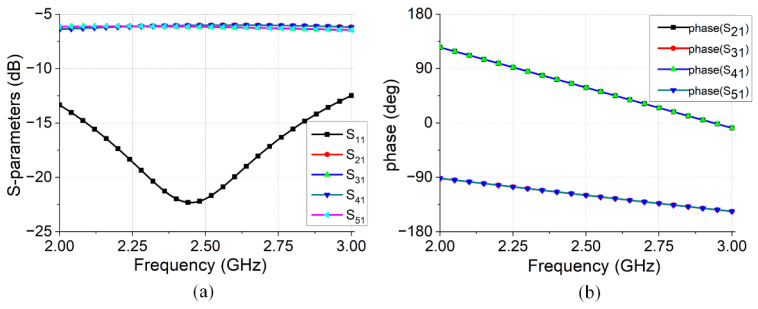
Simulation results for the feed network: (**a**) amplitude; (**b**) phase.

A specialized feed network structure was designed to meet the amplitude and phase requirements of each excitation port. The feed network ensures that each port is excited with equal amplitude and a 180° phase difference between adjacent ports. The simulated results for the feed network are shown in Figure 11. Within the 2.0–3.0 GHz operating frequency band, the input port maintains low S11 values, indicating good impedance matching. At 2.45 GHz, the insertion loss of the output port is 0.14 dB, and as Figure 11b shows, the phase difference between adjacent output terminals is approximately 180°, meeting the design requirements.

To study and analyze the influence of the antenna parameters on the performance of the antenna radiation patterns, the influence of the size of the metal ground plate and the length and width of the phase delay line of the feed network on the performance of the antenna were analyzed.

**Figure 12:**
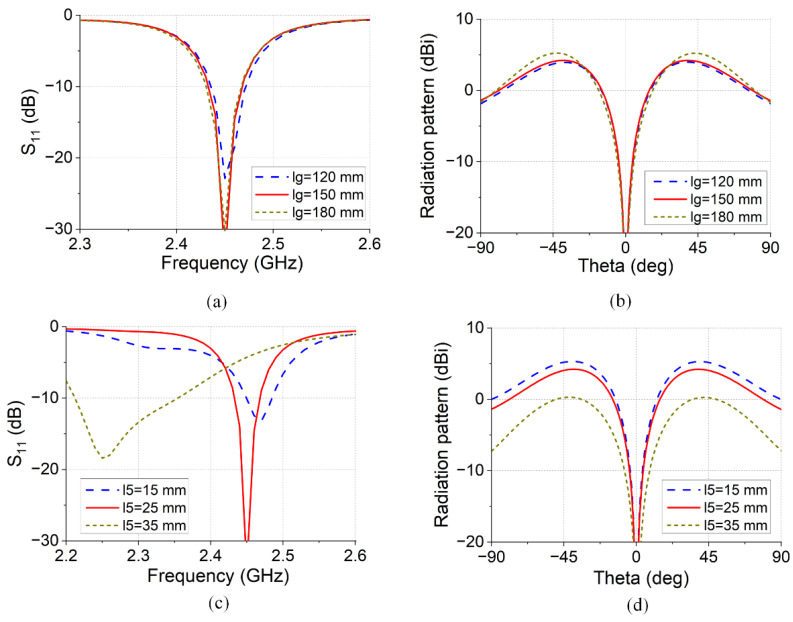
Antenna parameters analysis: (**a**) S11 variation in terms of ground size; (**b**) gain variation in terms of ground size; (**c**) S11 variation in terms of delay line length; (**d**) gain variation in terms of delay line length.

From Figure 12a,b, it can be determined that while the size of the antenna ground is changed from 120 mm to 180 mm, the operating frequency of the antenna is changed slightly, which shows that the size of the metal ground plate will affect the matching of the antenna, and with the increase in the size of the metal ground plate, the gain of the antenna will gradually increase. However, with a further increase in the size of the metal ground, the improvement space of the antenna gain is smaller.

From Figure 12c,d, it can be determined that, considering that the antenna works under the condition of a feeding signal with (0°, 180°, 0°, 180°), when the phase delay line is different, the length of the phase delay line changes from 15 mm to 35 mm, the resonant frequency of the antenna decreases from 2.48 GHz to 2.35 GHz, and the matching of the antenna becomes poor. At the same time, with the length of the delay phase line increase, the gain of the antenna changes from 4.5 dBi to 2.6 dBi at 2.45 GHz.

**Figure 13:**
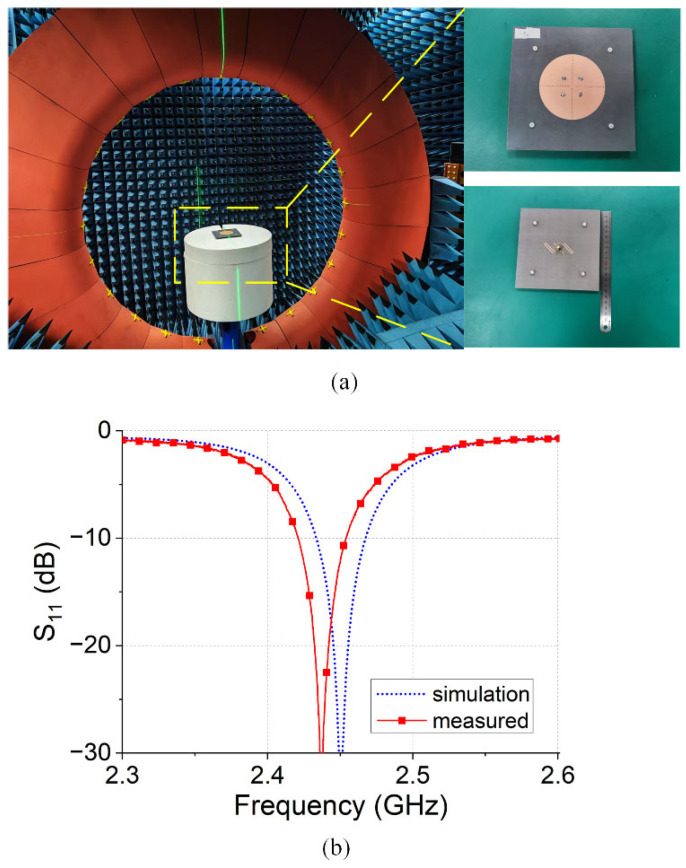
Antenna measurement: (**a**) measurement platform; (**b**) S_11_.

The simulation results for the array antenna are shown in Figure 13. The −10 dB bandwidth of the antenna is 2.42–2.45 GHz (30 MHz bandwidth), with an S11 value of −10.2 dB at 2.45 GHz.

**Figure 14:**
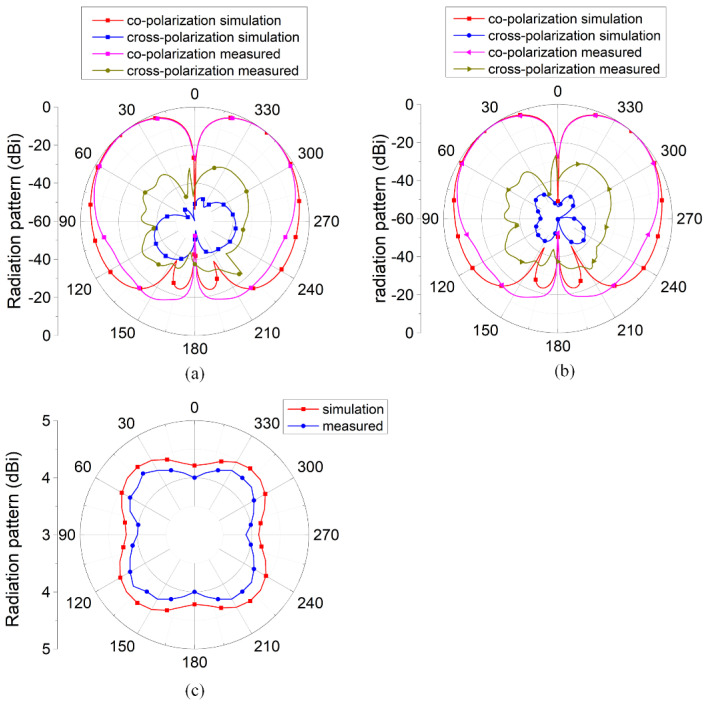
Radiation pattern of the antenna: (**a**) XOZ plane; (**b**) YOZ plane; and (**c**) peak gain at theta = 39°.

The radiation patterns of the antennas are illustrated in Figure 14. At 2.45 GHz, the antenna exhibited omnidirectional radiation characteristics in both the XOZ and YOZ planes, achieving a peak gain of 4.4 dBi.

A comprehensive performance comparison between the proposed omnidirectional antennas and previously published designs is presented in Table 1. The proposed antennas achieve peak gains of 4.1 dBi and 4.4 dBi, respectively. With a profile height of 0.028 λ_0_, the proposed designs exhibit a lower profile than similar antennas reported in the literature [13,15]. Additionally, the vertical polarization of the proposed antennas enables operation in metal-grounded environments, with a larger gain compared to those in the literature [14,16,17], enhancing their suitability for practical applications.

## 5. Conclusions

This study presents two distinct methods for the design of omnidirectional antennas, each employing a different feeding technique. The first method utilizes equal amplitude and in-phase excitation across all ports, whereas the second method employs equal amplitude, with a 180° phase difference between adjacent ports. The experimental results demonstrated that the two omnidirectional antennas achieved operating bandwidths of 2.45–2.58 GHz and 2.42–2.45 GHz, with gains of 4.1 dBi and 4.4 dBi at 2.45 GHz, respectively. Both designs exhibited high gain and low-profile characteristics. These omnidirectional antennas have promising prospects for applications in wireless energy harvesting.

## Figures and Tables

**Table 1:** Performance comparison of omnidirectional antennas.

Reference	Bandwidth (GHz)	Polarization	Peak Gain (dBi)	Size (λ_0_^3^) λ_0_@2.45 GHz
[13]	1.7–2.79	vertical	4.5	0.26 × 0.26 × 0.086
[14]	1.7–3.54	horizontal	0.6	1.23 × 1.23 × 0.0086
[15]	1.62–2.75	vertical	4.7	0.62 × 0.62 × 0.77
[16]	1.67–2.73	horizontal	1.7	0.98 × 0.98 × 0.0058
[17]	1.7–5.0	vertical	3.2	0.36 × 0.36 × 0.49
Antenna I	2.45–2.58 (5.3%)	vertical	4.1	1.12 × 1.12 × 0.028
Antenna II	2.42–2.45 (1.2%)	vertical	4.4	1.2 × 1.2 × 0.028

## Data Availability

The original contributions presented in the study are included in the article, further inquiries can be directed to the corresponding author.
